# An Alternative Cure: The Adoption and Survival of Bacteriophage Therapy in the USSR, 1922–1955

**DOI:** 10.1093/jhmas/jry024

**Published:** 2018-10-12

**Authors:** Dmitriy Myelnikov

**Affiliations:** Centre for the History of Science, Technology and Medicine, University of Manchester, Simon Building, floor 2, room 2.68, Manchester, M13 9PL, United Kingdom

**Keywords:** bacteriophage, Soviet medicine, antibiotics, microbiology, disease ecology

## Abstract

Felix D’Herelle coined the term bacteriophage in 1917 to characterize a hypothetical viral agent responsible for the mysterious phenomenon of rapid bacterial death. While the viral nature of the “phage” was only widely accepted in the 1940s, attempts to use the phenomenon in treating infections started early. After raising hopes in the interwar years, by 1945 phage therapy had been abandoned almost entirely in the West, until the recent revival of interest in response to the crisis of antibiotic resistance. The use of phage therapy, however, persisted within Soviet medicine, especially in Georgia. This article explains the adoption and survival of phage therapy in the USSR. By focusing on the Tbilisi Institute of Microbiology, Epidemiology and Bacteriophage (now the Eliava Institute), I argue that bacteriophage research appealed to Soviet scientists because it offered an ecological model for understanding bacterial infection. In the 1930s, phage therapy grew firmly imbedded within the infrastructure of Soviet microbiological institutes. During the Second World War, bacteriophage preparations gained practical recognition from physicians and military authorities. At the dawn of the Cold War, the growing scientific isolation of Soviet science protected phage therapy from the contemporary western critiques, and the ecological program of research into bacteriophages continued in Georgia.

“Enlisting Viruses As Allies To Fight ‘Superbugs’” – “Viruses Are The Antibiotics Of The Future” – “Viruses Save A Man From Antibiotic-Resistant Bacteria” – These headlines from the recent science media engage with what had long been an obscure medical intervention.^1^ In our age of anxiety over ever-expanding antimicrobial resistance and few commercial incentives to design new antibiotics, interest in historical alternatives has re-emerged. Bacteriophages, viruses that infect bacteria, offer hope of highly specific and efficient treatment for bacterial infection with few side effects and a low potential for resistance. These phages, as they are called, currently represent a means of last resort for severe infections, but they resonate with the growing ethos of personalized medicine. Clinical trials for wider use are in progress in Europe and the United States. Yet, this recent interest in phage therapy marks one end of a curious historical trajectory. Abandoned in the West in the 1940s, phage therapy had long persisted in the Eastern Bloc: Poland, Russia and especially Georgia.^2^

To a historian of biology, bacteriophages are most familiar as a key model in molecular biology, with origins in the Caltech “Phage Group” centered around Max Delbrück.^3^ Bacteriophages were instrumental in establishing DNA as the molecule of heredity through the Hershey-Chase experiment. They provided an early model for genetic structure, employed by the French microbiologist André Lwoff at the Pasteur Institute as a way to explain gene regulation. Yet, before World War II and the advent of electron microscopy, the viral nature of the phage was a subject of bitter debate, intensified by feuds over its discovery.^4^ “Bacteriophage” (usually translated from Greek as “devourer of bacteria”)^5^ was coined by the Canadian bacteriologist Félix d’Herelle in 1917 to describe both the phenomenon of the spontaneous clearing of cloudy bacterial cultures and the hypothetical agent behind this process, which d’Herelle believed to be a virus or a small microbe that could pass through the finest bacteriological filters. The English bacteriologist Frederick Twort had described a similar phenomenon in 1915. Opponents of d’Herelle in France and Belgium, who believed bacteriophages to be bacterial enzymes, fueled the priority dispute between the two.^6^ The bacteriophage proved a fascinating subject for microbiologists, manifesting a form of life at the boundary with nonliving molecules, with a dual life cycle that included a dormant lysogenic phase and an active lytic phase in which the phage rapidly destroyed bacterial cells.

Yet from the earliest discovery of the bacteriophage, therapeutic applications went hand-in-hand with the theoretical investigations, and d’Herelle himself used phage to treat dysentery, plague, and cholera. In the 1930s and 1940s, bacteriophage products were commercially available in France, Britain, Germany, Italy, and the United States. In a world before antibiotics, the use of phage was one of the many possible interventions against infection, and the sites for production ranged from reputable sources to questionable cottage industries. For various nation-specific reasons, phage therapy declined in most western countries during World War II, shortly before the triumph of penicillin.^7^ By contrast, phage therapy persisted in the USSR, even though the Soviets had established mass antibiotic production by 1950.

In what follows, I focus on the therapeutic uses of phages and their unique trajectory in Soviet medicine. Why did phage therapy appeal to Soviet medicine, and why did it persist in the USSR? I will argue the reasons are threefold. First, from the early days in the 1920s, phage became an active research topic, both as a theoretical problem and as a possible therapy. An ecological vision of disease prominent in interwar Soviet microbiology underpinned the scientific interest in phages, as did the emphasis on the links between theory and practice. These links showed synergy with the grandiose expansion of Soviet microbiology institutes that combined research, trials, and mass production. Phage research became especially entrenched in Georgia where a dedicated bacteriophage institute was founded in 1935. Second, the war efforts in the Winter War with Finland (1939–1940) and in World War II mobilized phage as a key therapy for dysentery, wound infections, and as prophylaxis against cholera. Finally, the rapid isolation of Soviet medical research in the early days of the Cold War meant that western dismissals of phage therapy were not taken seriously east of the Iron Curtain, and phage therapy could function as an alternative or a companion treatment to penicillin and other antibiotics. While phage research did decline across the USSR during the Cold War, scientists in Georgia maintained the therapy project alongside more basic research on bacteriophages and ensured its survival.

Today, the George Eliava Institute in Tbilisi is a key site dedicated to phage therapy. Known under various names throughout its history, it now carries that of its founder Giorgi (George) Eliava (1892–1937) who worked closely with d’Herelle. With a few exceptions, little has been written about the Soviet story of phage research and therapy. William Summers and David Shrayer-Petrov have discussed d’Herelle’s visits to Georgia in 1933–35, and science journalists Anna Kuchment and Thomas Häusler have written about phage therapy with a keen eye to its Soviet history.^8^ Nina Chanishvili at the Eliava Institute has done much to preserve its history and summarize the scientific heritage of Georgian phage work for an international audience.^9^ These works have described the wide use of phage therapy from the 1920s to the 2010s in diseases ranging from dysentery to typhoid. I wish to expand these accounts by elaborating the story of the institute’s origins and its scientific choices, theoretical commitments, and political maneuverings after Eliava’s execution during the Great Terror. I draw on previously unexamined archival sources, especially the papers of the Eliava Institute’s precursors and of the Georgian SSR People’s Commissariat of Health. I also expand the geographic scope of the existing story and show the great spread and diversity of phage research across the USSR during the Stalin years.

Furthermore, I argue that phage research and therapy were framed within an ecological vision of disease. Interactions between phages, bacteria, and humans were a key focus for Soviet phage researchers, especially in Georgia. Recent historical work has highlighted disease ecology as an important and overlooked tradition within microbiology, and one with curious Soviet influences.^10^ Within the context of the war effort, physicians attempted to use phages as broad-spectrum, ersatz versions of sulfa drugs or antibiotics, but postwar studies have paid much closer attention to the specific targeting of phages to bacteria, passaging them through patients and combining antibiotic treatments with phage therapy. In this sense, Soviet phage therapy represents an alternative drug trajectory to the familiar narrative that speaks to the dominance of chemical medicines.^11^ Within the idiosyncratic ecology of Soviet medical research bacteriophages found their niche.

## Early Soviet reception of bacteriophage and its ecological framings

In the summer of 1915, at the peak of World War I, a group of soldiers stationed at Maisons-Laffitte on the outskirts of Paris suffered from a severe and atypical outbreak of dysentery. D’Herelle, associated with the Institut Pasteur, was charged with a bacteriological investigation of these cases. Working with fecal samples, d’Herelle observed that the cultures of the as-yet-unknown bacterium would lyse and clear after a while; moreover, if drops of the cleared cultures were transferred into other cultures, they would clear them, too. Filtering the resulting solution to remove any bacteria did not stop the mysterious antimicrobial action. D’Herelle published his observations in 1917 proposing that lysis was caused by an “invisible microbe antagonistic to the dysentery bacillus.”^12^ He coined the term “bacteriophage” to refer to both the putative microbe that caused lysis, and the lysis phenomenon itself. After the discovery, d’Herelle dedicated the remainder of his nomadic career to work on this phenomenon.

Shortly after publication, other scientists claimed they had described similar phenomena before d’Herelle. English military bacteriologist Frederick Twort, who published similar observations in 1915, was a key contender. D’Herelle’s scientific opponents, Jules Bordet and Andre Gratia at the Pasteur Institute in Brussels, initiated a bitter priority dispute between d’Herelle and Twort, which persisted throughout the 1920s. While d’Herelle continued to present bacteriophage as an “ultramicrobe,” i.e. a virus, Bordet argued that it was an auto-catalytic bacterial enzyme. Others have been included in a retrospective reassessment of the discovery with reported observations going as far back as the late nineteenth century, which include notes by Ernest Hanbury Hankin, a colonial bacteriologist in British India. Another claimant, who became important for Soviet researchers, was Nikolai Gamaleia,^13^ a prominent Russian-Ukrainian microbiologist who had reported spontaneous lysis of plague cultures in 1898.

D’Herelle’s first description of bacteriophage appeared in the most cataclysmic year in Russian history. Yet after the two revolutions of 1917, the brutal civil war and “war communism,” the new Soviet state started a project of reconstruction. Science and medicine played an important role in the process. In the 1920s, Bolsheviks courted wary scientists and expanded patronage for research. The early Soviet leadership valued the sciences as key to building communism, industrializing the vast empire, educating the proletariat, and contributing to the materialist Marxist-Leninist ideology. Old institutions and university departments were revived and expanded, and new research institutes (*nauchno-issledovatel*′*skie instituty*, NIIs), free of undergraduate teaching, were being established. In the early days of Soviet power, there was a diversity of sponsors for the sciences. With Stalin’s takeover and the 1928 turn from New Economic Policy towards planned economy, scientific patronage was heavily centralized, and science was mobilized to serve the policy of the regime. The Soviet republics had their own, local arrangements that largely replicated the central model on a smaller scale. Most medical research remained the prerogative of the central and republican People’s Commissariats of Health, or *narkomzdrav*s.^14^

With the mass expansion of healthcare and the creation of socialized medicine in the 1920s, fighting infectious disease was a priority for the young Soviet state. Building on pre-Revolutionary research and capacities, including Pastorian and anti-plague stations, Soviet authorities invested in epidemiological control and surveillance. Prophylaxis of infection became a key goal linked to preventative epidemiological measures and speedy intervention at outbreak sites. An expanding network of the Institutes of Microbiology and Epidemiology was established in the capitals of the Soviet republics and other major cities. These institutes combined practice-oriented medical research and epidemiological studies with industrial production of vaccines, sera, and novel therapies, while also training medical practitioners.^15^

Bacteriophage received considerable interest from Soviet scientists, and some were in direct contact with western luminaries. D’Herelle’s first monograph, the 1921 *Bacteriophage: Its Rôle in Immunity,* was translated to Russian in 1926, followed in the next year by the work of his disciple Paul Hauduroy, *Le Bactériophage de d'Hérelle* (Paris: Librairie Le François, 1925).^16^ In two ways, the bacteriophage was more than a fashionable subject. First, its therapeutic promise suited both the practical ethos and the growing infrastructure of Soviet microbiological research. Second, d’Herelle’s controversial, but thrilling theories about the bacteriophage’s role in human immunity appealed to Soviet interest in symbiosis and an ecological vision of infection.

Institut Pasteur and its associated global networks served as a model for building Soviet bacteriology. Some of the key figures of Russian microbiology had links to Paris and had spent time there. The Nobel laureate and cellular immunity pioneer, Il′ia Mechnikov (Élie Metchnikoff), started his career in Odessa, but spent his later years in France. For Russian bacteriologists, he represented a direct link with the Pastorian tradition and achieved an almost iconic status with many institutes named after him. Lev Tarasevich, the first director of the Institute for Vaccine and Serum Control in Moscow, built links with the French Pastorians after the revolution, and organized high-level Soviet celebrations for the centenary of Pasteur’s birth in 1922.^17^ A similar link emerged for bacteriophage work in the figure of Giorgi Eliava, a Georgian scientist who had worked with d’Herelle in Paris in 1918–1920, and on his return headed the bacteriological laboratory in Tiflis (Tbilisi). Eliava had a direct connection to ground-breaking phage work, which was strengthened on his second visit to Paris in 1925–26. As d’Herelle’s ally, he was also an early and consistent proponent of the viral theory of bacteriophage. Yet Eliava was not the only adopter and champion of phage work in the USSR. Many other hubs in the growing microbiological network pursued bacteriophage research, especially in Moscow, Leningrad, and Kharkov (Ukrainian Kharkiv).

D’Herelle’s early work emphasized the interconnectedness between bacteriophage, bacteria, and the infected organism, and, controversially, proposed a role for the bacteriophage in immunity. He posited that animals had adopted phage as a weapon against infections alongside the established humoral and cellular immunity.^18^ Further, he suggested that during a pandemic or an epizootic, phages spread through a population in parallel with bacteria and were responsible for the eventual decline of an outbreak. D’Herelle thus framed epidemics in symbiotic terms.^19^ Alfred Tauber has argued that d’Herelle was “one of the few scientists of the period to have explicitly framed his ideas within the Mechnikovian worldview,” i.e. an evolutionary and symbiotic framework.^20^ Mechnikov suggested that phagocytes had been simple ancestral cells recruited by animals to fight foreign bodies – a view similar to d’Herelle’s perspective on bacteriophages.

Given the importance of Mechnikov’s work and image to Soviet bacteriologists, d’Herelle’s controversial theories were stimulating. Many figures engaged with them even if they did not accept his position. After the revolution, Nikolai Gamaleia resumed his 1898 experiments with bacterial lysins, and proposed bacteriophages as a subgroup among many agents that could break up bacterial cells. He suggested that bacteriophages were neither a virus nor an enzyme, but a shrunken bacterium that could pass through bacteriological filters – a hypothesis he abandoned in the 1930s.^21^ Leopold Peretts in Leningrad, who extended Mechnikov’s thinking on the importance of gut microflora and advocated a therapy based on *Bacillus coli* (now *E. coli*), suggested that phages might occasionally aid infection by destroying the protective bacteria; his work was later used to adjust a medicinal phage mix to protect gut bacteria.^22^ The Moscow-based microbiologist Lev Zil′ber, in his 1928 monograph on “paraimmunity” against microbes accompanying the causative agent of infection, engaged with d’Herelle’s phage model of immunity.^23^ At the Stavropol Anti-Plague Station, Magdalina Pokrovskaia investigated the role of phage in the complex host-parasite relation of bubonic plague. She isolated potent plague bacteriophages from the bodies of dead ground squirrels that carried fleas associated with the spread of the disease.^24^

The Soviet ecological perspective on the interaction between bacterial species and their hosts predated western interest in disease ecology.^25^ It was influenced, among other sources, by soil microbiology, especially Sergei Vinogradsky’s work on the life cycles and ecological understanding of microbial communities.^26^ The bacteriophage debates reached beyond medical bacteriologists as the new phenomenon inspired speculative theorizing. In 1927, the visionary earth scientist Vladimir Vernadsky wrote about bacteriophages on a cosmic scale, a year after the publication of his seminal *The Biosphere.* Vernadsky’s concept of the biosphere, influential in geography and climate science, posited life on Earth as a thin but complex and interconnected layer of the planet akin to the atmosphere, which has the ability to shape the planet alongside other geological forces. As he put it: life had a unique “geochemical energy.” Accepting d’Herelle’s virus hypothesis for the purposes of his argument, Vernadsky saw bacteriophage as the smallest and most liminal unit of life, which due to its size had the fastest velocity of “life transfer” in the biosphere. In this velocity, which Vernadsky estimated to exceed the speed of sound, as well as the omnipresence of phages, he saw proof of life’s constant pressure to expand its domain over inorganic matter.^27^

“D’Herelle’s phenomenon” thus inspired a diverse set of experimental programs in speculation in Soviet Russia, much as it did elsewhere in Europe. In 1933, Sof′ia Kazarnovskaia at the Leningrad Pasteur Institute published the first Russian monograph on bacteriophage, aimed at a wide audience of biologists and medical students.^28^ She focused on the arguments for both the bacterial enzyme and viral microbe hypotheses, and drew liberally on French and German literature. Without choosing a side, she did suggest that bacteriophage was a “substance with features of a creature,” an intermediate agent between life and inanimate matter.^29^ Her book also emphasized the medical promise of bacteriophages, one that appealed greatly to the growing microbiological community in the Soviet Union. By the time the book was published, first trials of medicinal uses of the phage had already begun in Soviet Ukraine.

## First trials: phage therapy in the Donbass

The mysterious phenomenon of the bacteriophage and d’Herelle’s provocative theorizing appealed to the Russian school of bacteriology, which was receptive to ecological thinking about disease and eager to discover connections between microbes, hosts, and immunity. Yet the practical aspect of bacteriophages was even more attractive to the expanding network of the Institutes of Microbiology and Epidemiology, whose mission was to combine research, evaluation of new therapies, and the production of “bacterial preparations,” such as vaccines. By that point d’Herelle and others had been developing phage therapies against dysentery, typhoid, plague, and cholera. Some of this work took place in Europe, notably Germany, but much happened elsewhere, in Egypt, India, French Indochina, and Brazil. D’Herelle himself went to Alexandria and then Mumbai to examine his theories of immunity in the practical setting of an active cholera epidemic.^30^ Inspired by these ventures, early trials of phage therapy and prophylaxis began in Soviet Ukraine in 1929, centered around Kharkov. These trials emphasized local needs and the importance of matching of bacteriophages to bacterial agents found in situ, while also seeking to create mixtures that would be efficient against several strains – exposing the tension between specificity and mass use that would come to distinguish Soviet phage therapy.

In 1886 the Kharkov Mechnikov Institute was established as a bacteriological station in Kharkov, a prominent center for medical education.^31^ During the Civil War, Kharkov was a hub for the Red Army, and after the briefly independent Ukraine fell, the capital remained there until Kiev (Kyïv) regained the title in 1934. Kharkov also neighbored the Donbass region, whose heavy industry and extensive coal mining made it a key center of early Soviet industrialization.^32^ Beginning in the late 1920s, workers migrated in large numbers into the region – most came from Russia, and epidemics were common. The Institute’s Vasyl′ Derkach had isolated the local Kharkov dysentery phage as early as 1922, one of the earliest such experiments in the USSR. In 1929, Moisei Mel′nyk and his colleagues initiated a program of testing phages for the therapy and prophylaxis of dysentery.

**Fig. 1.**
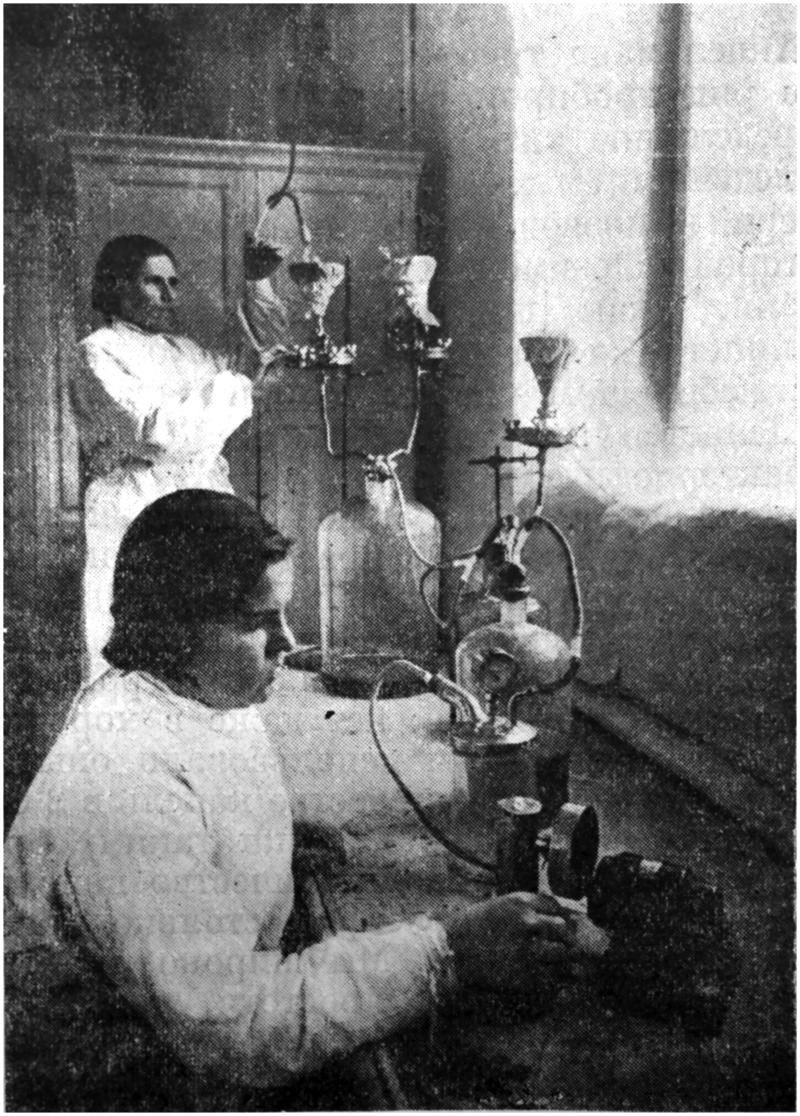
Technicians at the Kharkiv Mechnikov Institute filtering bacteriophage from lysed bacterial cultures. From Moisei Mel'nik and R. I. Hastovich, *Bakteriofag pri dizenterii* (Kharkiv: Gosudarstvennoie Meditsinskoe Izdatel'stvo, 1935): 44.

Between 1929 and 1935, the Kharkov team made regular expeditions to the Donbass region where they found frequent outbreaks of scarlet fever, typhoid and dysentery.^33^ They built strong connections with the hospitals in Stalino (now Donets′k), Alchevsk, Rykovo, and Krasnyi Luch, and collaborated with local physicians. The team tried several therapies, including two types of typhoid vaccine, a scarlet fever serum, and a bacteriophage against dysentery. The phage was made against the Shiga strain of the dysentery bacillus (now classified as *Shigella dysenteriae*) typical in the Donbass. Following d’Herelle, Mel′nyk isolated bacteriophages from local waters – the river Donets, and a sump (*emsher*) for treating sewage in Izium. He added the water samples to various Shiga substrains cultured form patients’ feces, grown in agar, and transferred to a medium based on meat stock. The phages would lyse the bacteria, turning the cloudy culture clear. At this stage, technicians would mix the resulting liquid with other cleared cultures and pass them through a bacterial filter that would trap bacteria and debris, but allow the phage through (Fig. 1). This approach achieved several goals. It raised local phages against local strains, achieving great specificity. The subsequent mixture offered a mix of phages active against various Shiga substrains resulting in a “polyvalent” phage that could treat a diverse set of local patients.^34^

Physicians in smaller Donbass hospitals performed most of the treatments. They reported on the cases to Mel′nyk, who pooled the results to create a total of 282 treated patients with 1059 controls where no phage was given. Mortality, while uncommon for dysentery, was halved in phage patients, and the four patients who died after phage therapy had been treated very late in the course of the illness. Recovery came quicker with phage treatment with fifty-five percent discharged within four days, compared to nineteen percent of control cases. The trials were thus promising, but not foolproof, and Mel′nyk designed new regimens. Under his guidance, physicians delivered oral phage preparations with soda water to reduce the stomach acidity believed to interfere with the treatment. Physicians were instructed to administer phages as early into the course of the illness as possible. Responding to their feedback, Mel′nyk insisted on complete fasting by patients on the day the phage was first given as well as the subsequent observance of a strict diet. In hypertoxic cases he recommended combining phage therapy with injections of antidysentery serum. While the latter was thought to fight the toxins released by bacteria, it did not always work on its own, and often caused immune reaction. The key advantage of phage therapy, on the other hand, was not only its apparent efficiency, but also a lack of side effects.^35^

For Mel′nyk, these first trials were largely successful and established some of the key parameters for future uses. Phage therapy had to be administered early to be most effective; it had to be tailored to the bacterial strains present in the epidemic region; and although a polyvalent mix had practical advantages in that it could help more patients, it still had to be based on local strains. Encouraged by these results, Mel′nyk expanded the scope of his work to phage prophylaxis of dysentery in children by testing single doses of the polyvalent phage when “summer diarrhea” was expected in hot weather. Furthermore, with the administration of the Mechnikov Institute, Mel′nyk continued building networks with physicians, public health authorities and other microbiologists via the hierarchy of the Institutes of Microbiology and Epidemiology. In 1934, via the Ukrainian Narkomzdrav, the Kharkov team established links with Eliava, who was by then running the Bacteriological Institute in Tbilisi. The two institutes committed to exchange bacterial cultures and phages, as well as research plans and reports of medical applications.^36^ In 1932 Mel′nyk’s collaborator from the Donbass expeditions, Hnat Ruchko, took over the management of the Mechnikov Institute, having recently returned from a two-year visit at the Research Institute of Hygiene and Immunology in Berlin, a hub for phage research in Germany. Ruchko sent his colleagues to pursue advanced degrees elsewhere in the USSR, and, at the tail end of the early Soviet policy of elevating national cultures and languages, set up a Ukrainian *Microbiological Journal.*^37^

In 1934 Ruchko moved to Kiev to head the new Institute of Microbiology of the Ukrainian Academy of Sciences. There, in 1936, he organized a major Ukrainian conference on “Bacteriophage and Microbe Variation,” which drew together microbiologists from the Ukrainian SSR and much farther throughout the Soviet Union. In total, seventy-eight papers were given, and 301 delegates attended. The sessions devoted to bacteriophages focused on both the nature of the phenomenon and its medical uses. The conference resolution preserved some ambiguity regarding the nature of the phage, even though most delegates agreed it came from bacteria in some form, and emphasized the need to purify it better for physical and chemical analysis. Yet when it came to medical applications, the resolution called for “its timely introduction into practice.”^38^

The conference in Kiev cemented the importance of the bacteriophage for Soviet microbiology, and the strong link between theoretical and practical investigations embedded in the institute system. The publication of the conference proceedings, however, was delayed until 1939, for tragic reasons.^39^ During Stalin’s Great Terror of 1937, both Mel′nyk and Ruchko were arrested and executed as enemies of the people and members of the “Right-wing Trotskyite organization in Ukraine.” Among the wildly preposterous, yet typical allegations meticulously listed in the NKVD files, the two were accused of “conducting sabotage in microbiology using the preparations of bacteriophage, making preparations for bacteriological warfare” and recruiting microbiologists unhappy with Party policy.^40^ Their names had to be rapidly erased from the publication of conference proceedings as well as subsequent reviews and histories. As I discuss below, a similar fate awaited Eliava in Tbilisi.

## Building institutions: All-Union Institute “Bacteriophage”

While Kharkov and Kiev had the potential to become key sites for phage therapy, the persecution of their leaders and the subsequent evacuation of staff before the Nazi occupation of Ukraine meant that little momentum remained for this work. Yet a different site dedicated to phage research had emerged by that point. The Tbilisi Institute of Microbiology, Epidemiology and Bacteriophage (IMEB) had been authorized in 1935 and opened in 1939. Its charismatic founder, Giorgi Eliava, drove its creation with deft administrative skill by building on his close connections with d’Herelle as well as navigating the treacherous landscape of Soviet scientific patronage. While Eliava also perished in the terror, the institutional support that he had cultivated would nurture phage therapy after his execution and through the war.

Between 1933 and 1935, d’Herelle came to Georgia twice for extended visits to work with Eliava at the Bacteriological Institute of the People’s Commissariat of Health (*Narkomzdrav*) of the Georgian SSR in Tiflis (the Georgian name, Tbilisi, was adopted internationally in 1936). He had left behind his position at Yale, after conflict over research directions and money, to be welcomed as a guest of honor. *Pravda* covered his second arrival, describing d’Herelle as “one of the most outstanding microbiologists in Western Europe.”^41^ While in the USSR, he toured the key scientific centers including Kharkov and Moscow, and attended microbiology conferences in Baku and Leningrad. In Moscow, Grigorii Kaminsky, the People’s Commissar of Health for the Russian SFSR, offered d’Herelle to pick a relevant institute in the capital that could be devoted to the problem of the bacteriophage. D’Herelle refused, driven by his links to scientific workers in Tiflis, but also citing the benefits of the warm Georgian climate for his respiratory ailments.^42^

While in Georgia, d’Herelle completed his survey work, *Bacteriophage and the Phenomenon of Recovery*, which was translated to Russian by Eliava and published by the Tiflis University Press in 1935.^43^ Naturally, it opened with a dedication to Stalin, who was described as someone who, “driven by the unconquerable and merciless logic of history, builds human society on completely new principles.”^44^ There is some debate about d’Herelle’s exact political views, and the dedication could well have been rubber-stamped without his knowledge. But he certainly had sympathy for the Soviet state, a sentiment made sharper by witnessing the Great Depression in the United States, a period he recalled with vivid sadness in his autobiography. Another passage from his foreword is perhaps more indicative: “I have written [this book] for the scientists of the USSR, this wonderful country which, for the first time in history, did not choose irrational mysticism as its guide, but sober science.”^45^

Busy and excited with his celebrated guest, Eliava sought to put this collaboration on a firmer institutional footing, and recruited patrons for a brand-new institute in Georgia. Backed by Pëtr Agniashvili, the deputy head of the Transcausacian Sovnarkom, Eliava appealed to Lavrentiy Beria, then secretary of the Transcaucasian Committee of the Communist Party. The Transcaucasian Sovnarkom had committed 200,000 roubles of the 940,000 required, but the remainder was still to be secured. In his letter to Beria, Eliava emphasized d’Herelle’s reputation and the practical uses being made of phage, both in Georgia and globally. He stressed the wide use of phages against bacillary dysentery in the USSR and in Brazil; that plague phages like the ones he and d’Herelle had isolated were being tested in Egypt and Madagascar; the great potential of phages against the so-called “war diseases” (*voennye infektsii*) – typhoid and paratyphoid – which had taken more lives than the actual fighting; and the new developments of phages for treating wound infections, again with a clear military relevance.^46^

**Fig. 2.**
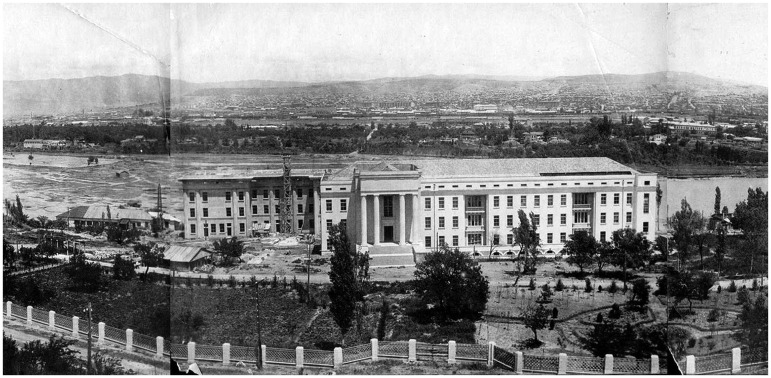
Construction of the Tbilisi Institute of Microbiology, Epidemiology, and Bacteriophage on the bank of the Kura river in Saburtalo, Tbilisi, c. 1939. Courtesy of the National Parliamentary Library of Georgia, Digital library “Iverieli;” owner: Nikoloz Abashidze.

Beria did not oblige; and though the 1920s, an era of strong patronage for the sciences, had now passed, there were ways around the obstacles. While Georgia’s relatively peripheral status could have been a problem, several Georgian Bolsheviks had played key roles in the revolution, and a few still carried considerable weight. Eliava appealed to Sergo Ordzhonikidze, the People’s Commissar of Heavy Industry and a Politburo member.^47^ In April 1936, the Sovnarkom of the USSR approved the construction and the release of funds, and the USSR Narkomzdrav vetted a building project in December 1936, with construction beginning the same month.^48^ The new institute was being built on the bank of the river Kura in Saburtalo, then the outskirts of Tbilisi, on the site of the Bacteriological Institute’s stables where serum-producing horses were kept. The Leningrad architect Fëdor F. Berenshtam designed the new building in a neoclassical Stalinist style, with a portico and two parallel buildings connected through a passage (Fig. 2). On the grounds, a large “French cottage” was planned for d’Herelle’s and Eliava’s families. When d’Herelle moved back to Paris in late 1935, he intended to return.

But then came the Great Terror. On 22 January 1937, Eliava was arrested in his house on accusation of anti-Soviet activity, one of the early victims in the year of the Great Terror. Much speculation exists about the motivation of this arrest, including a feud with Beria. Eliava was accused of espionage for France, and was used as one of the witnesses in the case against an old Bolshevik, Budu Mdivani. As the terror avalanched, new accusations involved sabotaged vaccines and poisoning wells with bacteriophage. Eliava was executed on 10 July 1937.^49^

After Eliava’s death, the fate of the Bacteriophage Institute was in peril, but the plans had already been approved at the Sovnarkom level, and considerable funds had already been spent, so the building work carried on, with slight modifications.^50^ In May 1937, the All-Union Sanitary Inspection suggested a merger between the planned bacteriophage institute and the existing Bacteriological Institute. As a result, the original name, All-Union Institute “Bacteriophage,” was dropped, and the combined body became the Research Institute of Microbiology, Epidemiology and Bacteriophage (IMEB), now under the control of the Georgian Narkomzdrav. This merger was approved by the USSR Narkomzdrav in 1938, and construction resumed and was almost complete in 1939, and staff moved into the new building that year.^51^ Razhden Korchilava was appointed director, while the scientific council guided research policy. Korchilava had only completed his medical training two years prior to this promotion, but unlike his more established colleagues, he had been a Party member since 1921, which trumped his lack of scientific clout for this largely managerial position.^52^

The new institute combined research, therapeutic trials, epidemiological studies and production of “bacterial preparations” (*bakpreparaty*). It was split into microbiological, epidemiological, serum, vaccine and phage departments; a separate anaerobic department for wound treatment and bacteriophages against wound infections; a small biochemical department; a brucellosis laboratory; and separate units for smallpox and tuberculosis (BCG) vaccines. There was also an auxiliary culture media unit, and a reference collection of living bacterial cultures. While other microbiology institutes in the USSR also pursued phage research, only the Tbilisi IMEB maintained a dedicated bacteriophage department.

**Fig. 3.**
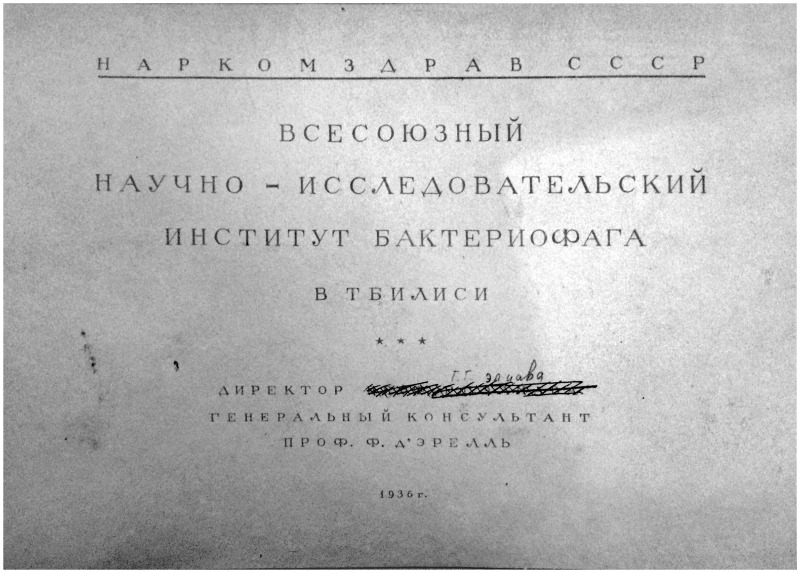
Architectural plans for the All-Union Bacteriophage Institute, frontispiece. Prof. F. d'Herelle is listed as the consultant general, and the director's name, G. G. Eliava, is meticulously erased. It has since been written in by hand, perhaps by the librarian. Courtesy of Nina Chanishvili and the George Eliava Institute of Bacteriophages, Microbiology and Virology, Tbilisi.

In typical Stalinist fashion, Eliava’s name was erased from his institute, sometimes literally so (Fig. 3). Whatever institutional memory of Eliava remained after his death, it did not survive in written records. Like most arrested during the terror, he had his identity suppressed, even after his rehabilitation in 1956. It was not until the late 1960s that his name began to be mentioned again. Yet, several of his former collaborators remained in the new IMEB. Vladimir Antadze was relocated from Tbilisi Medical University as permanent consultant. Alexander Tsulukidze, a practicing surgeon who had worked with Eliava and d’Herelle, collaborated closely with the new institute. Most significantly, Elena Makashvili, who had been Eliava’s assistant since the early 1920s, headed the bacteriophage department. As of 1939, the institute declared several research programs on bacteriophage in its scientific plan, most continuous with the work under Eliava. They researched phage mixes, or “polyphages,” which could be used as broad-spectrum interventions as well as new methods for mass production, especially preservatives for liquid phage products. Some work engaged with d’Herelle’s ideas, especially when it came to prophylaxis. Prescribing the dysentery bacteriophage against the Shiga-Kruse bacillus during local epidemics in nearby villages, and attempting to introduce phages into local wells, Makashvili and her colleagues pursued d’Herelle’s speculation that individuals could spread the phage through the population and keep infections under control.^53^ Nikolai Abashidze, the institute’s head of production, also researched bacteriophage ecology both in the environment (wells and the river Kura) and in the bodies of healthy and diseased people.^54^

Eliava’s fate, along with wild accusations of poisoning wells reminiscent of Nazi propaganda, could have shut down bacteriophage research in Georgia and beyond, but the promise of phage research and therapy persisted. While the plans for the initial All-Union institute were scaled down, phage research and therapy became cemented in a new research establishment, which exists to this day. Shortly after the IMEB began operating, the military needs of the Soviet Union pushed phage therapy to the forefront of applied microbiology. As the USSR invaded Finland in 1939–1940, and then joined the Allies after Nazi Germany invaded in June 1941, medical trials of bacteriophages expanded.

## Bacteriophages at war

With new infrastructure much had been invested in phage. The Tbilisi IMEB opened in 1939, but its ambitious production plans were unrealistic amid continuous building works. More established institutions took the initiative. The same year, Zinaida Ermol′eva, a senior microbiologist at the flagship All-Union Institute of Experimental Medicine (VIEM) in Moscow, appealed to the Scientific Council of the Soviet Narkomzdrav to expand bacteriophage research and production capacities. In her report, Ermol′eva stressed the uncertainty over the nature of the bacteriophage. While she did not believe it was a virus, she proposed further biochemical studies to help understand its nature. She also emphasized the successes of phage therapy, noting that “phage therapy of dysentery gained recognition among physicians.”^55^ In cases of cholera, the situation appeared more urgent. With outbreaks in China and Afghanistan, therapy and prophylaxis were of great importance – indeed, Ermol′eva’s team had been experimenting with phage treatment of wells at the border with Afghanistan in 1938, and found that more active polyvalent phages against cholera were needed. The expansion of production was also about securing Soviet borders.^56^

Ermol′eva’s solution was to establish a new laboratory at VIEM to study phage biochemistry; to organize production of cholera phage in Moscow and Tbilisi; to improve the overall quality of phage production, following d’Herelle’s criteria; and to catch up with western competitors. The last point appeared to refer to scientists in Europe, but she also may have alluded to the extended industrial production of phages by the German pharmaceutical firm Behringwerke that started earlier that year.^57^

Ermol′eva concluded:

We hope that the in-depth approach enabled by the [important] role of science in the Soviet Union, will allow for the expansion of bacteriophage use in the practice of Soviet socialist healthcare. Moreover, I have no doubt that the practical uses will aid in solving a number of theoretical problems.^58^

**Fig. 4.**
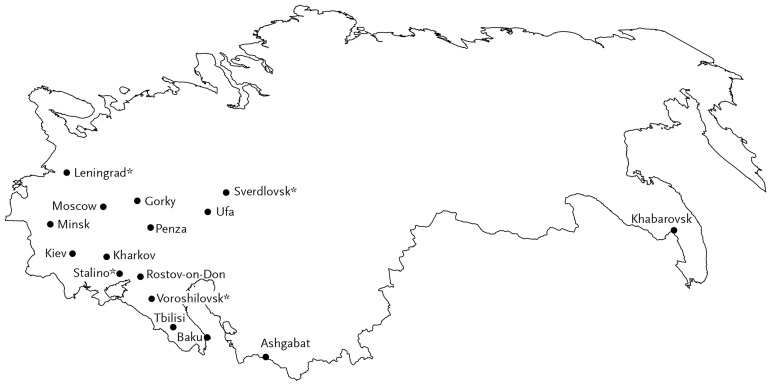
Map of phage research in USSR, c. 1940, based on conference contributions and journal articles. * Names are given as of 1940: Leningrad is now St. Petersburg, Sverdlovsk is Ekaterinburg, Stalino is Donets'k, and Voroshilovsk is Stavropol'.

The Narkomzdrav Scientific Council responded with cautious enthusiasm. All agreed on the importance of bacteriophage research – the Leningrad biochemist Vladimir Englegardt was the most emphatic, calling it an “exceptional problem” and a major case for “the unity of theoretical and practical work.”^59^ But views on phage therapy varied. Nikolai Budrenko, the Surgeon General of the Red Army, had used phages against staphylococcus infection with largely poor results. The eminent pediatrician Georgy Speransky cited failures of phage therapy in children, noting he had “a whole crate of bacteriophage from Ukraine” that did not work.^60^ Ermol′eva’s response was to promote better production of phage through the standardization of methods, thorough testing of samples, and the use of polyvalent phages – this strengthened her case for a new laboratory at VIEM. Despite skeptical voices, the scientific council decided to invest in phage work, calling for the expansion of Ermol′eva’s lab, setting up production of cholera and dysentery phages, as well as pyo- (wound infection) and intesti-phage mixes. By 1940, when the next major conference on bacteriophages took place in Moscow, a large proportion of microbiological institutes pursued some form of research into phage therapy, including places as remote as Khabarovsk in the Russian Far East and Ashgabad in Turkmenistan (Fig. 4).

The military importance of phages was tested in the early 1940s. In August 1939, the USSR and Germany signed the Molotov-Ribbentrop pact, with the secret protocol dividing Eastern Europe between the two countries. A few weeks after German armies breached the Polish border on 1 September 1939, the USSR invaded Poland from the east. After a rapid and successful incursion, the Red Army started another campaign in the end of November, this time against Finland. What followed was the Winter War, and it proved a disaster for the Soviet side. Despite a three-fold military advantage, the Red Army met sustained and unexpected resistance and a series of defeats. In March 1940, the Moscow Peace Treaty was signed, whereby Finland ceded some of its territory adjacent to Leningrad, but maintained its sovereignty.

For surgeons, the Finnish war was an opportunity to evaluate new medical tools, including bacteriophages for anaerobic wound infections that led to gangrene. With Leningrad less than thirty miles from the frontline, and a prominent scientific center, the Sanitary Service of the Red Army assigned Magdalina Pokrovskaia, who had moved from Stavropol to Leningrad, to test phages on the wounded. Her group was joined by Alexander Tsulukidze’s team of surgeons from Tbilisi – the same Tsulukidze who had worked with Eliava and d’Herelle on staphylococcus and streptococcus phages.^61^ After taking over a twenty-five-bed ward of the Leningrad Red Army Hospital, the team soon moved to an evacuation hospital closer to the frontline where it worked in a 120-bed surgical department.^62^ Using the phages made in Tbilisi and Moscow, the bacteriologists tested their efficacy against their patients’ specific infecting microbe *in vitro,* and the surgeons administered them as part of a wound treatment regimen.

Tsulukidze and Pokrovskaia’s work received backing from senior army physicians, even if fellow bacteriologists were not always convinced. Thus, at a meeting of the Leningrad Pirogov Surgical Society, Leopold Peretts doubted the efficiency of the *Bacillus perfringens* phage that the team used in fighting anaerobic infection.^63^ Professor Ponomarëv of the Leningrad Institute of Vaccines and Sera suggested phage effects *in vitro* could not be assumed to happen in patients, and recommended future trials that would incorporate a serum his group had developed. In response to the skepticism, the deputy head of the Red Army Sanitary Service, Pëtr Zhuravlëv, who had worked with Tsulukidze and Pokrovskaia, chided the Leningrad surgeons for their conservative attitudes. If anything, he claimed, the phage team was too modest and cautious, which may have “damaged state interests.” Unequivocally, he declared,

If our Leningrad medical community, the veteran surgeons, tackle this question properly, by the time the next wars are imposed on us we will have a powerful medicine with which we will be victorious against all infected wounds.^64^

After the Finnish war, Tsulukidze continued to promote wound phages, with growing military and scientific support. Zhuravlëv’s military superiors, Efim Smirnov and Nikolai Burdenko, were early proponents and patrons. On Tsulukidze’s return to Tbilisi, the Georgian Narkomzdrav took interest in this work, channeling all patients with infected cuts or wounds into his clinic. Eventually, Tsulukidze was decorated with the Order of the Red Star for his work.^65^ In 1940, the Georgian Narkomzdrav set up a bacteriophage committee within its Scientific Council. The phage committee, which included Tsulukidze, Makashvili and Antadze, was charged with monitoring research plans, proposing new topics for investigation, and popularizing phage among physicians and surgeons by publishing protocols and guidelines. Tbilisi IMEB started mass production of dysentery phage and the intestiphage mix, as well as some wound phages, despite the struggles with incomplete building works and lack of meat supplies for culture media.^66^ While the researchers may have promoted specificity of treatment, which would involve culturing wound bacteria and matching the phages to them, in practice production focused on making generic polyvalent mixes of phages that could be used against a broad range of strains.^67^

Yet it was not only the microbiology institute infrastructure that produced phages. One advantage of this therapy was the ability to set up local production with modest means. All that was required were phage samples that could be obtained from local water or patients, meat-based media, and cultures of pathogens. Several stories of local production during WWII reflect this utility. In the summer of 1942, Georgy Miterev, the People’s Commissariat of Health sent Ermol′eva to Stalingrad (now Volgograd), a major city on the frontline in Southern Russia, where the key battle would happen later that year. At that stage, Stalingrad was a military hub with major army traffic, and after some local cases of cholera, sanitary authorities anticipated a pandemic. Ermol′eva made it to Stalingrad on a small plane, but German bombers destroyed the freight train that carried phages from Moscow. Ermol′eva had to set up her own production, handling local cholera vibrios in a hospital basement in order to prepare for subsequent mass prophylaxis.^68^ Similarly, cholera and dysentery bacteriophages were made in the besieged Leningrad under haphazard conditions.^69^

Patients’ perspectives on bacteriophages are difficult to reconstruct, but memoirs and diaries offer a glimpse, particularly those written by citizens of Leningrad during the Siege (September 1941–January 1944). In conditions of extreme starvation, “hunger diarrhea” was a common diagnosis, and Leningrad pathologists attributed most cases to dysentery.^70^ Contemporary entries and later recollections suggest dysentery bacteriophages were in short supply and of great value. Thus, on 9 April 1942, Irina Zelenskaya, an economist at the Seventh State Power Station, reflected on her relationship with the machinist Kashtanov, ill with colitis, whom she “visited… in the hospital all the time, and now I help him out a lot, to the extent that I got him some bacteriophage.”^71^ Boris Strugatsky, perhaps the most celebrated Soviet science fiction author alongside his brother and co-author Arkady, recalled of the Siege:

Then, in March, I came down with the so-called bloody diarrhea, an infectious disease that is dangerous even for a grown, portly man, and I was eight years old, and had dystrophy – a certain death, one would think. But our neighbor (who also miraculously survived) somehow happened to have a vial of bacteriophage, so I lived.^72^

These views of phage work contrasted with the opinion of physicians in Britain and the USA, and their wartime experience. In a systematic 1941 review of various trials in the *Journal of the American Medical Association,* Krueger and Scribener found no consistent case for phage efficiency.^73^ British military physicians Boyd and Portnoy tested a German-made dysentery phage in Egypt, on Italian prisoners of war, with poor results despite high phage activity *in vitro*.^74^ Towards the end of the war, collaboration between the USSR and Western Allies was at its peak, and medical exchanges prominent. In the winter of 1943–44, a small Anglo-American delegation of scientists departed for Moscow. On the US side, the delegation included the biochemist Albert Baird Hastings and Michael Shimkin, a Russian emigre cancer researcher who also acted as the interpreter. The UK Medical Research Council (MRC) sent Howard Florey, the driving force behind mass production of penicillin, and Arthur G. Sanders as his assistant. Their mission was to establish contacts with Soviet physicians, and the conversation covered measures in cases of chemical or biological warfare, new surgical techniques, and – crucially – penicillin.

Reporting back on the work on phage therapy at VIEM, Florey remained skeptical: “It is impossible to estimate what this phage work amounts to. Much of it, I feel, belongs to the ‘Impressionist’ school of science”.^75^ The British readers of the report, which the MRC circulated, did not express much interest in phage either; thus, Alan Drury, the director of the Lister Institute of Preventative Medicine, wrote that his colleagues agreed that “until the ‘phage’ or details of its preparation were available, none of them were prepared to pay much attention to it.”^76^

Soviet physicians were much more enthusiastic about the work on phages, but not unanimous in their support, either. Retrospectively, Ermol′eva explained her motivation to pursue penicillin as a response to the inefficiency of anaerobic phages in cases of sepsis.^77^ In a postwar, thirty-one-volume magnum opus, *The Experience of Soviet Medicine in the Years of the Great Patriotic War,* phage therapy for dysentery was described as disappointing; wound phages had better reviews, but sulfa drugs and penicillin were received with far greater enthusiasm.^78^ While phages had served an important role in the Soviet war efforts, other medicines could have replaced it after the war, as they did elsewhere.

## Survival: Antibiotics, phage and the early Cold War

Bacteriophages existed alongside other drugs and interventions, and the emphasis on containing outbreaks through strict sanitary surveillance. Thus, for dysentery, microbiological institutes also produced a vaccine following the Institut Pasteur’s Besredka method.^79^ There had been minor production of sulfa drugs before the war, but more became available from the Allies from 1942, and these were used alongside bacteriophages, sometimes as a combined treatment.^80^ Penicillin and a wave of other antibiotics, notably Soviet gramicidin S, soon became the major new hope for fighting infections, making phage less relevant. While there were shortages and production difficulties, penicillin had become largely available to Soviet physicians by 1950. Yet the new drugs did not make phages obsolete. Soviet researchers maintained phages within a larger repertoire of biological agents to fight infections. Moreover, the institutional infrastructure in Tbilisi, and the tenacious commitment of its scientists to the problem of the bacteriophages, created a niche for this therapy. The close connection between theoretical work on phage, new uses for typing bacterial strains in diagnosis, and studies of bacterial variation, and clinical investigations carried phages through the difficult political terrain of late Stalinism.

Penicillin was an important part of the USSR’s partnership with the Allies in the last phases of World War II, and played its part in the detente of the early Cold War. As Nikolai Krementsov has argued, scientific issues were key to 1940s diplomacy, and these went beyond the Bomb and included biomedical research.^81^ Anglo-American war efforts transformed penicillin from a curious substance discovered by Alexander Fleming in 1929 to a wonder drug. Building on the Oxford research of Florey and Ernst Chain on isolating the substance from *Penicillium notatum,* the US Office of Scientific Research and Development coordinated mass production of penicillin through deep fermentation. By 1943, penicillin was being made for military and some civilian uses on a large scale in both the USA and Britain.^82^ Soviet medical and military authorities followed the Ally developments with great interest. In parallel with her work on phage prophylaxis, Zinaida Ermol′eva was assigned to run the Soviet penicillin project. It is likely she first learned about the drug from Florey’s publications, but there have been plausible suggestions that the Soviets learned about the serious efforts around the drug through espionage.^83^ While the Allies supplied the USSR with sulfa drugs via lend-lease, they were less forthcoming with penicillin. Ermol′eva and her group started investigating local mold samples, and eventually managed to isolate sufficient quantities of distinct penicillin, which they called *krustozin* or “Penicillin VIEM,” for early trials on wounded soldiers.^84^ During Florey’s visit to Moscow, the Soviet authorities presented the collaborative penicillin trials as a friendly competition between the two preparations, with Russian krustozin showing much higher efficiency (Florey’s report offers a dramatically different version).^85^ After the war, the USSR invested in penicillin. Krustozin, however, did not prove amenable to mass production, and the deteriorating relations with the USA hindered attempts to secure production rights for the western methods. Eventually, the Soviets bought a penicillin license from Ernst Chain as one of the patent holders.^86^

The start of the Cold War affected Soviet science in drastic ways. International collaboration encouraged during the war years became nearly criminal “adulation [*nizkopoklonstvo*] of Western science.” The fight against “rootless cosmopolitanism” (meaning international outlook, but routinely a euphemism for being Jewish) escalated in 1946–47, and science had to be purged of western links. On 17 March 1948, *Pravda* announced that “penicillin is a Russian discovery,” citing both Ermol′eva’s work and obscure nineteenth-century mold researchers.^87^ In 1949, Vil Zeifman, the man in charge of mass production of penicillin, came under heavy criticism for insisting on securing western penicillin rights when Russian krustozin should have been used. During the late-Stalinist anti-Semitic campaign Zeifman was fired and subsequently arrested. After the investigators’ interrogation methods led to a heart attack, he was exiled to Siberia.^88^

It was in this context that phage therapy persisted, in growing isolation from western science and its dismissals of this treatment. While phage was no longer the major attraction for therapeutic uses, and prominent scientists like Ermol′eva looked elsewhere, research continued in more peripheral Soviet Georgia. After the war, Tbilisi IMEB no longer had to spend all resources on production, and research work renewed. Demand for some bacteriophage products – the cholera phage, for instance – declined, but the institute continued making dysentery, intesti- and pyo-phages, and expanded its portfolio into products against typhoid. The institutional synthesis of basic microbiological research, clinical investigations and production of biologics allowed the group to persist in using phage clinically.

After the war, most Soviet literature abandoned d’Herelle’s ideas about the crucial role of phages in the extinction of epidemics and the enhancement of human immunity. The ecological focus of phage research, however, remained attractive to Soviet microbiologists and persisted in a holistic research program in Tbilisi. In the report on IMEB’s bacteriophage research in 1946–49, the head of the bacteriophage department and Eliava’s protégée Elena Makashvili envisioned the institute’s mission in such terms:

Developing this problem as a whole, and considering the living nature of bacteriophage, the Institute bases its work on the principle of an unbreakable bond between the living organism and its environment, which becomes especially apparent when developing questions around the study of bacteriophages that show pronounced variability depending on environmental conditions, [i.e.] living microbes (in a given medium), for which bacteriophage is in turn a potent factor of variation.^89^

The Bacteriophage department pursued several lines of research after the war. Makashvili’s report admitted certain limitations in the use of phage therapy in dysentery, mostly stemming from the general ignorance of how phage behaved inside the human body, but presented this challenge as a case for further work. Similarly, she did not see the new antibiotics as a reason to abandon phage therapy. Much like Ermol′eva’s group at VIEM had investigated joint use of phage with sulfa drugs, Tbilisi scientists pursued experiments that combined phage therapy with penicillin and gramicidin treatments. Their results suggested that antibiotics did not diminish phage activity, and sometimes combined therapy had greater efficacy. Similarly, in Leningrad, Moisei Fisher, who had taken over the microbiology department at the Sanitary-Hygienic Medical Institute, promoted phage therapy and integrated work on phage, antibiotics, and antibodies, presenting them as a spectrum of what he called “biological antiseptics.”^90^ While greatly appreciated by Soviet scientists, physicians and medical authorities, antibiotics were not necessarily magic bullets, but rather parts of a broader arsenal. By contrast, in the US, between 1945 and 1955, the focus on broad-spectrum antibiotics and antibiotic mixes, driven by pharmaceutical companies, obscured other interventions, and concerns over resistance only emerged later.^91^

As bacteriophage was becoming a prominent model in molecular biology, IMEB sent staff to Moscow to train in using radioisotopes, but the local biochemical expertise proved insufficient. On the other hand, the specificity of the relationship between phages and bacteria was recruited in phage typing – i.e. using bacteriophages to distinguish between substrains of bacteria, aiding in diagnosis and microbial classification, especially for salmonellae and brucellae. The work had a more ambitious focus on bacterial variation more generally as Teimuraz Chanishvili examined how phages adapted to mixed cultures of salmonellae, drawing conclusions about evolutionary relationships between these bacteria.^92^

Despite the interest in bacterial variation, scientists at the institute did not couch their research in genetic terms. This would have been a dangerous avenue to pursue as Lysenkoism took over Soviet biology after the infamous 1948 meeting at the Lenin All-Union Academy of Agricultural Sciences (VASKhNIL). After the meeting, Nikolai Bystry, a junior scientist who joined IMEB after the war, delivered a talk on “Michurinist microbiology” and called for, among other things, new studies on bacteriophage adaptation to develop heritable heat resistance.^93^ “Michurinist microbiology” did not resurface in IMEB’s discussions beyond this talk, and may well have been lip-service. In any case, IMEB’s environmental focus allowed its scientists to present its work within a Lysenkoist framework with minimal effort.

In 1949, the Soviet Ministry of Health^94^ reviewed IMEB’s work favorably, and envisioned a bigger role for the Institute in Transcaucasia. It did, however, criticize the state of phage research, urging more work on the nature of phages, as well as the prophylaxis of dysentery and typhoid. IMEB workers were also chided for ideological lapses, especially important during the early Cold War, and were warned against “adulation of bourgeois science” and giving much credit to foreign scientists. For example, the Ministry’s representative comrade Sokolov stressed, “Everyone now knows that our scientist Gamaleia discovered the bacteriophage phenomenon, whereas this gets attributed to d’Herelle.”^95^ This atmosphere of isolation from western science, while going against Eliava’s ethos, had at least one benefit. The institute workers could continue investigating bacteriophages with support from the Soviet state, and the western dismissals of phage therapy did not pose a real challenge.

## Conclusion

In 1955, the Tbilisi Institute of Vaccines and Sera (as the IMEB became in 1953) called a major conference on bacteriophage, bringing workers from across the USSR. In an introductory talk, Vladimir Antadze triumphantly summarized the recent electron microscopy and radioisotope evidence that redeemed d’Herelle’s original hypothesis – that phages were viruses. He also stressed what had been at stake when it came to pursuing phage research over the previous decades: “Even in the most difficult times for the problem of bacteriophage, our institute maintained its original focus to the best of our ability, with the tenacity of a ‘lysogenic strain.’”^96^ This tenacity, compared to the dormant stage in the phage life cycle, paid off. As phage therapy persisted in the postwar years, Tbilisi scientists sought to become an officially recognized hub for bacteriophage research. In 1953, they had successfully lobbied the Ministry of Health to become the methodological center for work on bacteriophages across the USSR, which meant they directed the work of approving and disseminating production standards and clinical regimens, but also coordinating research plans in other institutes. Phage therapy was thus provided with an even stronger institutional backing, alongside more theoretical investigations.

The cementing of phage therapy in Tbilisi was a result of decades of institution building, but also several contingencies. Soviet medical research provided a unique environment for the treatment to persist. In its early years, phage therapy appealed to the ecological orientation of many Soviet microbiologists, and while they did not always accept d’Herelle’s provocative models of the phenomenon, his work was a source of inspiration. The network of microbiological institutes that combined research with mass production made phage therapy an attractive pursuit, reinforced by international links with leading proponents of the treatment. Despite the devastating effect of Stalinism on some of the key actors, local schools persisted, and the dramatic military needs in the Winter War and the Great Patriotic War sustained the demand for phage therapy.

After the war, while much attention and funding was diverted to penicillin and other antibiotics, phage therapy survived in a niche in Georgia. While the growing isolation of Soviet science in the early years of the Cold War was detrimental to much research, it also cushioned phage researchers from western dismissal and disinterest. Yet the relative isolation of phage research, as well as the overall inertia of the Soviet bureaucracy and the planned economy, can only partially explain the survival of this therapy. The local campaigning and persistence of scientists at the Tbilisi IMEB, their careful navigation of shifting political terrains, and an overall commitment to the value of bacteriophage were equally important.

## Funding

This work was supported by the Wellcome Trust (grant number 106639/Z/14/Z). I declare no conflict of interest.

## Footnotes

1 Jason Gale, “Enlisting Viruses as Allies to Fight ‘Superbugs’,” *Bloomberg Businessweek,* November 27, 2017; https://www.bloomberg.com/news/articles/2017-11-27/enlisting-viruses-as-allies-to-fight-superbugs-quicktake-q-a; Daniel Overhaus, “Viruses are the Antibiotics of the Future,” *Motherboard*, December 7, 2017, https://motherboard.vice.com/en_us/article/9kdbqa/bacteriophages-phage-therapy-antibiotic-resistant-bacteria; Liz Sockett, “Viruses Save a Man from Antibiotic-Resistant bacteria”, in “Laughing Parrots, Backflipping Robots and Saviour Viruses: Science Stories of 2017,” *Observer,* December 24, 2017, https://www.theguardian.com/science/2017/dec/24/early-man-microplastics-the-year-in-science.

2 On phage therapy, see William Summers, “Cholera and Plague in India: The Bacteriophage Inquiry of 1927–1936,” *J. Hist. Med. Allied Sci.* 48, no. 3 (1993): 275–301; Summers, *Félix d'Herelle and the Origins of Molecular Biology* (New Haven & London: Yale University Press, 1999); Summers, “Bacteriophage therapy,” *Ann. Rev. Microbiol.* 55 (2001): 437–451; Thomas Häusler, *Viruses vs. Superbugs: A Solution to the Antibiotics Crisis?* trans. Karen Leube (London: Macmillan, 2006); Nina Chanishvili, “Phage Therapy: History from Twort and d'Herelle through Soviet Experience to Current Approaches,” *Adv. Virus Res.* 83 (2012): 3–40.

3 On the Phage group, see Lily Kay, *The Molecular Vision of Life* (Oxford: Oxford University Press, 1993), 243–268; Michel Morange, *A History of Molecular Biology* (Cambridge, MA: Harvard University Press, 1998), 40–61.

4 Morange, *History of Molecular Biology:* 150–164. See also Neeraja Sankaran, “The Bacteriophage, Its Role in Immunology: How Macfarlane Burnet’s Phage Research Shaped His Scientiﬁc Style,” *Stud. Hist. Philos. Biol. Biomed. Sci.* 41 (2010): 367–375; Ton van Helvoort and Neeraja Sankaran, “How Seeing Became Knowing: The Role of the Electron Microscope in Shaping the Modern Definition of Viruses,” *J. Hist. Biol* (2018), published ahead of print 20 June 2018, https://doi.org/10.1007/s10739-018-9530-2.

5 d’Herelle took objection to the “devourer” translation, as his intended meaning was more “develop at the expense of,” but most science writing used the devourer metaphor -- Summers, *Felix d’Herelle*: 191–192.

6 Summers, *Félix d’Herelle*; on Twort, see Antony Twort, *In Focus, out of Step: A Biography of Frederick William Twort FRS, 1877–1950* (Stroud: Alan Sutton, 1993).

7 Emiliano Fruciano, “Phage as an Antimicrobial Agent: d’Herelle’s Heretical Theories and Their Role in the Decline of Phage Prophylaxis in the West,” *Can. J. Inf. Dis. Med; Microbiol.* 18 (2007): 19–26; Anna Kuchment, *The Forgotten Cure: The Past and Future of Phage Therapy* (New York: Copernicus Books, 2012): 35–42.

8 Summers, *Félix d’Herelle*: 161–172; Shrayer-Petrov, “Félix d’Herelle in Russia,” *Bull. Inst. Pasteur* 94 (1996): 91–96; Häusler, *Viruses vs. Superbugs*; Kuchment, *The Forgotten Cure*. See also “Under the Sign of Bacteriophage: Paris–Tbilisi”, *Science First Hand* 46, no. 1 (2017), https://scfh.ru/en/papers/under-the-sign-of-bacteriophage-paris-tbilisi, accessed 9 March 2018.

9 Nina Chanishvili, “Phage Therapy.” The review of Georgian phage work has been published in English as Chanishvili, *A Literature Review of the Practical Applications of Bacteriophage Research* (New York: Nova Biomedical, 2012).

10 Warwick Anderson, “Natural Histories of Infectious Disease: Ecological Vision in Twentieth-Century Biomedical Science,” *Osiris* 19 (2004): 39–61; Anderson, “Postcolonial Ecologies of Parasite and Host: Making Parasitism Cosmopolitan,” *J. Hist. Biol.* 49, no. 2 (2016): 241–59; Mark Honigsbaum, “‘Tipping the Balance:’ Karl Friedrich Meyer, Latent Infections, and the Birth of Modern Ideas of Disease Ecology,” *J. Hist. Biol.* 49, no. 2 (2016): 261–309.

11 Jean-Paul Gaudillière, “Introduction: Drug Trajectories,” *Stud. Hist. Philos. Biol. Biomed. Sci.* 36, no. 4 (2005): 603-611; see also other articles in that issue; Alexander von Schwerin, Heiko Stoffand & Bettina Wahrig, Introduction to *Biologics: A History of Agents Made From Living Organisms in the Twentieth Century* (London: Pickering & Chatto, 2013).

12 Summers, *Félix d’Herelle:* 41–59; “On an Invisible Microbe Antagonistic to Dysentery Bacilli. Note by M. F. d’Herelle, Presented by M. Roux. *Comptes Rendus Academie des Sciences* 1917; 165:373–5,” trans. Hans-W. Ackermann, *Bacteriophage*, 1 (2011), no. 1: 3–5

13 Russian, Ukrainian and Georgian names have been romanized here following the ALA–Library of Congress convention, but omitting diacritics and simplifying certain endings. All translations from Russian and Ukrainian are the author’s own.

14 Nikolai Krementsov, *Stalinist Science* (Princeton: Princeton University Press, 1997): 13–45; Frances L. Bernsetin, Christopher Burton and Dan Healey, “Experts, Expertise and New Histories of Soviet Medicine,” in *Soviet Medicine: Culture, Practices and Science* (DeKalb, IL: Northern Illinois University Press, 2010): 3–26.

15 Much work on the history of Soviet microbiology remains to be done, but see Nikolai Krementsov, *The Cure: A Story of Cancer and Politics from the Annals of the Cold War* (Chicago: University of Chicago Press, 2002): 33–51; Elizabeth Hachten, “How to Win Friends and Influence People: Heinz Zeiss, Boundary Objects, and the Pursuit of Cross-National Scientific Collaboration in Microbiology,” in Susan Gross Solomon, ed. *Doing Medicine Together: Germany and Russia Between the Wars* (University of Toronto Press, 2006): 159–198; Michael Z. David, “Vaccination against Tuberculosis with BCG,” in Frances L. Bernsetin, Christopher Burton and Dan Healey, eds. *Soviet Medicine: Culture, Practices and Science* (DeKalb, IL: Northern Illinois University Press, 2010): 3–26.

16 Feliks d’Erell′, *Bakteriofag i ego znachenie dlia immuniteta* (Moscow & Leningrad: Gosudarstvennoe izdatel′stvo, 1926); Pol′ Odiurua, *Uchenie o bakteriofagakh d’Herelle’ia* (Leningrad: Prakticheskaia meditsina, 1927). Most scientists trained before the revolution would have read them in French if they could access copies.

17 Hachten, “How to Win Friends and Influence People.”

18 Summers, *Félix d’Herelle*: 108–120.

19 Jan Sapp, *Evolution by Association: A History of Symbiosis* (Oxford: Oxford University Press, 1994): 106–108.

20 Alfred Tauber, *The Immune Self: Theory or Metaphor?* (Cambridge: Cambridge University Press, 1994): 87.

21 Nikolai Gamaleia & O. I. Shvetsova, “Issledovaniia o bakteriolizinakh i bakteriofagakh,” *Gigiena i epidemiologiia*, (1924) no. 1: 1–8; Nikolai Gamaleia, *Biologicheskie protsessy razrusheniia bakterii* (Moscow: Gosudarstvennoe izdatelʹstvo meditsinskoi literatury, 1934): 35–43.

22 Discussed in L. I. Nakhimson, “K voprosu o control mul′tivalentnykh fagov,” *ZhMEI* (1942), no. 3–4: 72–76

23 Lev Zilʹber*, Paraimmunitet* (Moscow: Izdatelʹstvo 1-go Moskovskogo Gosudarstvennogo Universiteta, 1928): 47–50; Lev Kiselëv and Elena Levina, *Lev Aleksandrovich Zil*ʹ*ber (1894-1966): Zhizn′ v nauke* (Moscow: Nauka, 2004): 111–121.

24 Ilʹia Benʹkovich, *13 let nauchnoi meditsiny na Severnom Kavkaze* (Rostov-on-Don: Severnyi Kavkaz, 1934): 50–51.

25 Anderson, “Natural Histories of Infectious Disease.”

26 Lloyd Ackert, *Sergei Vinogradskii and the Cycle of Life: From the Thermodynamics of Life to Ecological Microbiology, 1850–1950* (Heidelberg: Springer, 2013). On the discussion of Vinogradsky’s ecological analysis versus the pure culture method dominant in microbiology, see Mathias Grote, “Petri Dish versus Winogradsky Column: A Longue Durée Perspective on Purity and Diversity in Microbiology, 1880s–1980s,” *Hist. Philos. Life Sci.* 40 (2018): 11, https://doi.org/10.1007/s40656-017-0175-9.

27 Vladimir Vernadskii, “Bakteriofag i skorost′ peredachi zhizni v biosfere”, *Priroda* (1927), no. 6: 434–446. Jonathan Oldfield and Denis Shaw, “V.I. Vernadskii and the Development of Biogeochemical Understandings of the Biosphere, c.1880s–1968”. *Br. J. Hist. Sci.* 46, no. 2 (2013): 287–310.

28 Sofʹia Kazarnovskaia, *Bakteriofagiia* (Leningrad: Izdatel’stvo Akademii Nauk SSSR, 1933).

29 Ibid., 109. Russian words for substance (*veshchestvo*) and creature (*sushchestvo*) rhyme.

30 Summers, “The Bacteriophage Inquiry.”

31 “Ocherk istorii ukrainskogo instituta epidemiologii i mikrobiologii imeni Mechnikova,” *Trudy Ukrainskogo Instituta im. Mechnikova* 10 (1946), pp. 37–54; Moisei Mel′nik and Ignatii Ruchka [sic], “Spetsificheskaia terapiia dizenterii bakteriofagom,” *Vrachebnoe delo* (1930) no. 9, 3–7.

32 On the history of the Donbass, see Hiroaki Kuromiya, *Freedom and Terror in the Donbas: A Ukrainian-Russian Borderland, 1870s–1990s* (Cambridge: Cambridge University Press, 2003).

33 “Ocherk istorii ukrainskogo instituta,” 51–52.

34 Moisei Mel′nik and R. I. Hastovich, *Bakteriofag pir dizenterii* (Kharkov: Gosudarstvennoie Meditsinskoe Izdatel′stvo, 1935). The tension between “polyvalent” and “monovalent” treatments, i.e. mass use vs. specificity, were also prevalent in other microbiological interventions, such as serotherapy and subsequent development of antibiotics. See Scott Podolsky, *Pneumonia before Antibiotics: Therapeutic Evolution and Evaluation in Twentieth-Century America* (Baltimore: Johns Hopkins University Press, 2006): 18–25.

35 Ibid., 12–28.

36 National Archives of Georgia, Tbilisi (NAG), fond 289, opis′ 1, delo 1443.

37 Vira Hamaliia, “Hnat Omelianovych Ruchko (1883–1937): Trahichna dolia vchenogo,” *Mikrobiolohichnyi Zhurnal* 72 (2010), no. 3, pp. 65–74. On early Soviet nationalities policies, see Terry Martin, *The Affirmative Action Empire: Nations and Nationalism in the Soviet Union, 1923–1939* (Ithaca & London: Cornell University Press, 2001).

38 Hnat Ruchko, “Konferentsiia po bakteriofahiï i minlyvosti mikrobiv,” *Mikrobiolohichyi Zhurnal* 4 (1937), no. 1, pp. 185–188, quoted from p. 187

39
*Minlyvist′ mikrobiv i bakteriofahiia* (Kyïv: Vydavnytstvo Akademiï Nauk URSR, 1939).

40 “Sledstvennoe delo no. 123 antisovetskoi pravo-trotskistskoi organizatsii na Ukraine,” central KGB archive no. P-411737, courtesy of the archives of the Ukrainian Security Service (SBU); Hamaliia, “Hnat Omelianovych Ruchko”.

41 “V SSSR vtorichno priezzhaet professor d’Errel′,” *Pravda* 298, no. 6184, (28 October 1934), p. 6. On d’Herelle’s visits to Georgia, see Shrayer-Petrov, “d’Herelle in Russia”; Summers *Félix d’Herelle*, 161–172; Félix d’Herelle, *Autobiographie de Félix d’Hérelle: Les Pérégrinations d’un bactériologiste,* ed. Alain Dublanchet (Paris: Editions Médicales Internationales, 2017): 311–313.

42 Iraklii Georgadze, “Tbilisskii institut vaktsin i syvorotok MZ SSSR za 50 let,” in *Teoreticheskie i prakticheskie voprosy bakteriofagii* (Tbilisi: TNIIVS, 1974).

43 Félix d’Herrelle, *Bakteriofag i fenomen vyzdorovleniia (*Tiflis: Izdatel′stvo Tiflisskogo Gosudarstvennogo Universiteta, 1935). It was published in France three years later, as d’Herelle, *Le phénomène de la guérison dans les maladies infectieuses* (Paris: Masson, 1938).

44 d’Herelle, *Bakteriofag:* 2.

45 d’Herelle, *Bakteriofag*: 6. See also Häusler, *Viruses vs. Superbugs*: 134–135.

46 Georgadze’s transcript of NAG f. 617, op. 1, d. 7698, l. 75, kept at the Eliava Institute.

47 Georgadze, “Tbilisskii institut;” Shrayer-Petrov, “d’Herelle in Russia.” On Beria, see Amy W. Knight, *Beria: Stalin's First Lieutenant* (Princeton: Princeton University Press, 1993). On Ordzhonikidze, see Oleg Khlevniuk, *In Stalin’s Shadow: The Career of “Sergo” Ordzhonikidze* (Armonk, NY: M. E. Sharpe, 1995).

48 NAG f. 289, op. 1, d. 2652, l. 18–25; d. 3071, l. 23–26

49 Eliava’s wife, the operatic soprano Amelia Wohl-Lewicka, had been arrested with him and was executed shortly after. Their 21-year-old daughter Ganna was arrested in April, and eventually condemned to 5 years of labour camps in Kazakhstan as a “socially dangerous element”. I have been able to reconstruct the details of Eliava’s arrest and last days based on Ganna’s memoirs -- Eliava-Malieva, “Vospominaniia” *Russkii Klub* (2017), nos. 2–6 -- and on the copies of documents from Budu Mdivani case, kindly provided by the Archives of the Ministry of Internal Affairs of Georgia. Eliava’s own case file does not survive. See also Dmitriy Myelnikov, “Giorgi and Felix: Phage Therapy in Georgia (Part II),” *Multispecies Medicine* (blog), 5 August 2018, https://www.multispecies.org/copy-of-giorgi-and-felix-pt2, accessed on 15 August 2018.

50 State Archive of the Russian Federation (GARF) f. 5446, op. 18, d. 2808, l. 45–50.

51 NAG f. 289, op. 1, d. 2652, l. 18–25.

52 Such appointments were typical -- see Krementsov, *Stalinist Science*: 20.

53 NAG f. 1991, op. 1, d. 1.; NAG f. 1991, op. 1, d. 15.

54 NAG f. 289, op. 1, d. 2668, l. 1–4.

55 GARF f. 8009, op. 2, d. 247, l. 22.

56 Little is known about the work in Afghanistan, and even in her 1942 monograph on cholera that explicitly deals with phage prophylaxis, Ermol′eva is very vague as to the exact location. It is likely the work was classified. Zinaida Ermol′eva, *Kholera* (Moscow: Medgiz, 1942): 81.

57 Häusler, V*iruses vs. Superbugs*: 105–109.

58 GARF f. 8009, op. 2, d. 247, l. 28.

59 Ibid.*,* l. 41.

60 Ibid., l. 35.

61 Their work was summarised in M. P. Pokrovskaia, L. S. Kaganova, M. A. Morozenko, A. G. Bulgakova and E. E. Skatsenko, *Lecheniie ran bakteriofagom* (Moscow & Leningrad: Medgiz, 1941) and Alexander Tsulukidze, *Opyt primeneniia bakteriofaga v usloviiakh voennogo travmatizma* (Tbilisi: Gruzmedgiz, 1941).

62 Tsulukidze, *Opyt:* 334.

63 The official transcript of this meeting is reprinted in Tsulukidze, *Opyt*: 410–418.

64 Tsulukidze, *Opyt*: 416.

65 Sokrat Arshba, Dmitrii Murvanidze, and G. Kuparadze, *Aleksandr Petrovich Tsulukidze* (Tbilisi: Metsniereba, 1973).

66 NAG f. 289, op. 1, d. 3441.

67 Aleksandr Tsulukidze, *Kratkoe nastavlenie po primeneniiu bakteriofaga pri lechenii ran* (Tbilisi: Gruzmedgiz, 1942): 11–12.

68 Zinaida Ermol′eva, “Nezrimaia armiia,” in *Dlia pobedy v Stalingrade* (Moscow: Sovetskaia Rossia, 1973): 293–301; Liubov′ Zheltova, “Voennoe zdravookhranenie v Stalingradskoi bitve,” *kandidat nauk* thesis, Volgograd State Pedagogical University, 1999.

69 Fëdor Mashanskii, “V otvete za zhizn′ liudei,” in T. M. Golubeva and N. B. Vetoshnikova (eds.), *Mediki i blokada: vzgliad skvoz*′ *gody* (St. Petersburg: Mezhdunarodnaia assotsiatsiia blokadnikov goroda-geroia Leningrada, 1997): 9–36. Phages were also used in the Gulag for prophylaxis of intestinal infections among inmates, although it is not clear whether they were produced locally. Andrei Eizenberger, *Esli ne vuskazus*′*—zadokhnus*′ (Moscow: Vozvrashchenie, 1994), pp. 67–68, www.sakharov-center.ru/asfcd/auth/?t=book&num=693, accessed on 4 January 2018. On medicine in the Gulag, see Golfo Alexopoulos, *Illness and Inhumanity in Stalin’s Gulag* (New Haven: Yale University Press, 2017); Dan Healey, “Lives in the Balance: Weak and Disabled Prisoners and the Biopolitics of the Gulag,” *Kritika* 16, no. 3 (2015): 527–556.

70 Vadim Chirsky, “The Work of Civilian and Military Pathologists,” in John Barber and Andrei Dzenkievich (eds.), *Life and Death in Besieged Leningrad, 1941–1944* (Basingstoke: Palgrave Macmillan, 2005)

71 9 April 1942, Irina Zelenskaia diary, available at prozhito.org/person/640, accessed 5 January 2018.

72 “Lichnost′: Boris Strugatskii,” *Argumenty i Fakty,* 21 May 2001.

73 A. P. Krueger & E. J. Scribner, “The Bacteriophage: Its Nature and its Therapeutic Use,” *JAMA* 116 (1941): 2160–2167.

74 J. S. K Boyd & B. Portnoy, “Bacteriophage Therapy in Bacillary Dysentery,” *Trans R Soc Trop Med Hyg* 37 (1944):243-262; Kuchment, *Forgotten Cure*: 37–42.

75
*Report of the Scientific Mission to Moscow 1944 by H. W. Florey with the Assistance of A. G. Sanders,* p. 65, Royal Society Archives, London: Howard Florey papers, HF 1/3/13/2.

76 Drury to Mellanby, 20 April 1944, The National Archives (UK): FD 1/6789

77 Ermol′eva “Nezrimaia armiia:” 296.

78 Efim Smirnov (ed.), *Opyt sovetskoi meditsiny v Velikoi Otechestvennoi Voine, 1941–45 gg.,* vol. 2 (Moscow: Medgiz, 1951): 48; vol. 31 (1955): 201.

79 See Ilana Löwy “Biotherapies of Chronic Diseases in the Inter-War Period: From Witte’s Peptone to Penicillium Extract” *Stud. Hist. Philos. Biol. Biomed. Sci.* 36, no. 4 (2005): 675–695.

80 John E. Lesch, *The First Miracle Drugs: How the Sulfa Drugs Transformed Medicine* (Oxford: Oxford University Press, 2006): 217–219.

81 Nikolai Krementsov, “In the Shadow of the Bomb: U.S.-Soviet Biomedical Relations in the Early Cold War, 1944–1948,” *J. Cold War Stud.* 9, no. 4 (2007): 41–67.

82 Robert Bud, *Penicillin: Triumph and Tragedy* (Oxford: Oxford University Press, 2007): 35–50.

83 Yurii Zeifman, interviewed by Nikolai Gladkikh, “Penicillinovoe delo,” *Mezhdunarodnoe Obshchestvo “Memorial”,* nos. 93–94, 30 October 2010; available online at http://old.memo.ru/2010/02/04/penicillin.htm, last accessed 25 January 2018.

84 Tatiana Mel′nikova, *Skvoz′ zavesu nevidimogo* (Volgograd: Nizhne–Volzhskoe knizhnoe izdatel′stvo, 1984): 46–55.

85 The Soviet version of the episode was recounted in propaganda at the time, and more famously in Veniamin Kaverin’s *Otkrytaia kniga* (*The Open Book)*, a 1955 novel closely based on Ermol′eva life and work -- Kaverin was her brother-in-law through her first marriage to Lev Zil′ber. In the report to the MRC, however, Florey showed little enthusiasm for Soviet penicillin; he believed the small doses used could not work, and recounted that “these cases [patients treated with krustozin] we never saw although we asked to see them repeatedly. They had always been dressed earlier or something of that sort.” *Report of the Scientific Mission to Moscow:* 30.

86 Yuri Zeifman, “Penitsillinovoe delo”; Mauro Capocci, “Cold Drugs: Circulation, Production and Intelligence of Antibiotics in Post-WWII Years,” *Medicina nei Secoli Arte e Scienza* 26, no. 2 (2014):. 401–422; Bud, *Penicillin*: 75–96.

87 “Penicillin—russkoe otkrytie,” *Pravda* 17 March 1948: 3.

88 Yuri Zeifman, “Penitsillinovoe delo”.

89 NAG d. 1991, op. 1, d. 64, l. 119.

90 Moisei Fisher (ed.), *Biologicheskie antiseptiki: Bakteriofagi, antitela, antibioitiki* (Moscow & Leningrad: Gosudarstvennoe Izdatelstvo Meditsinskoi Literatury, 1950).

91 Scott Podolsky, *The Antibiotic Era: Reform, Resistance, and the Pursuit of a Rational Therapeutics* (Baltimore: Johns Hopkins University Press, 2014): 19–30.

92 NAG d. 1991, op. 1, d. 64, l. 121. The adaptation of phages to *Salmonella* strains was the subject of Teimuraz Chanishvili’s 1949 *kandidat nauk* dissertation at IMEB, “Granitsy adaptatsii i readaptatsii bakteriofagov palochek gruppy Salmonell.” Some of this work is discussed in Teimuraz Chanishvili, “K voprosu differentsiatsii osnovnykh serologicheskikh vidov sal′monell pri pomoshchi bakteriofaga” in *Bakteriofagiia: Sbornik trudov mezhinstitutskoi nauchnoi konferentsii, sostoiavsheisia v g. Tbilisi 26–29 oktiabria 1955 goda* (Tbilisi: Gruzmedgiz, 1957): 207–216.

93 After the breeder Ivan Michurin (1855–1935), who became a mascot of Lysenkoism and was retroactively recruited as his precursor. Bystry’s talk is in NAG f. 1991, op. 1, d. 29. For an overview of Lysenkoism, see Nils Roll-Hansen, *The Lysenko Effect: The Politics of Science* (Amherst: Humanity Books, 2005).

94 In 1946, all People’s Commissariats (narkoms) became “ministries.”

95 NAG f. 1991, op. 1, d. 35, l. 48.

96 Vladimir Antadze, “Osnovnye zadachi izucheniia problemy bakteriofagii i puti ikh resheniia,” in *Bakteriofagiia: Sbornik trudov mezhinstitutskoi nauchnoi konferentsii, sostoiavsheisia v g. Tbilisi 26–29 oktiabria 1955 goda* (Tbilisi: Gruzmedgiz, 1957): 5.

